# Knowledge, attitudes, and practices of safe drinking water, sanitation, and hygiene among pre-adolescent students in public and private schools in Jordan: cross-sectional school-based study

**DOI:** 10.3389/fpubh.2026.1925764

**Published:** 2026-09-08

**Authors:** Aya Abu Aqab, Thikrayat Majdi Yahya, Mahmoud Al-Hussami, Abeer Ismail

**Affiliations:** 1 Public Health PhD Program, Faculty of Graduate Studies, Al-Quds University, Jerusalem, Palestine; 2 Community Health Nursing, The University of Jordan, Amman, Jordan; 3 Public Health PhD Program, Faculty of Graduate Studies, Al-Quds University, Gaza, Palestine

**Keywords:** attitudes, hygiene, knowledge, practices, sanitation, school, water

## Abstract

**Background:**

Maintaining good hygiene, which is easily applied with access to safe and adequate water, is an important preventive step against various infectious disease transmissions that might spread as a result of unclean water and inadequate sanitation. Jordan is one of the world's most water-stressed countries, and only one in three schools has basic sanitary facilities, which poses severe health risks to students. Assessing WASH-related knowledge, attitudes, and practices provides valuable evidence to guide interventions that promote healthy behaviors and reduce these risks.

**Purpose:**

This study aimed to assess the level of WASH knowledge, attitudes, and practices among preadolescent schoolchildren and to examine the relationship between student knowledge, attitudes, and practices with selected socio-demographics, as well as to investigate differences between public and private schools' students in Jordan. Furthermore, identifying predictors of schools‘ pre-adolescent practices.

**Participants and Methods:**

A cross-sectional School-Based Study design was employed among a sample of School students from Amman city (*n* = 600). Data collection included a Self-administered questionnaire and a list of school characteristics that were observed and recorded. The statistical analysis included descriptives, Pearson r correlation, independent *t*-test, and hierarchical linear regression.

**Results:**

Statistics revealed a significant positive relationship (*p* < 0.001) between knowledge and attitude, and between (*p* < 0.001) knowledge, attitudes, and practices with grades and age. A *t*-test revealed a statistically significant difference between public and private schools in scoring attitudes and practices of water, sanitation, and hygiene (*P* ≤ 0.001, *P* ≤ 0.000, respectively). Despite the absence of significant differences in knowledge between public and private schools, six items had the highest agreement rate. Furthermore, a multiple hierarchical linear regression analysis showed that sex, school type, age, knowledge, and attitudes were significant predictors of practices (*p* < 0.001).

**Conclusion:**

The results of our study showed the need for interventions to address gaps and challenges in health education and to develop policies to ensure adequate, hygienic WASH facilities, particularly in public schools, to reduce health risks, especially among pre-adolescent age groups.

## Introduction

Access to safe drinking water and adequate sanitation and proper hygiene (WASH) are not just basic needs but fundamental human rights and essential components of public health (1). WASH is central to achieving the Sustainable Development Goals (SDGs) and to guaranteeing high-quality, inclusive, and equitable education (Goal 4). This education is a powerful tool for achieving other goals, including ensuring access to sustainable water and sanitation management (Goal 6) (2).

Despite the clear advancement in hygiene practices in most developing countries, poor hygiene practices are still observed in others (3–8), while global water and sanitation challenges remain a major concern in the 21st century. Approximately 2 billion people (26% of the population) live in water-scarce countries, and 3.6 billion (46%) do not have access to well-run sanitation globally (9, 10). This inadequate water, sanitation, and hygiene contributes to the overall mortality rate and Disability-adjusted life years (DALYs) in low- and middle-income countries (LMICs) (11). The COVID-19 pandemic highlighted the inadequate coverage of hand hygiene in LMICs worldwide, underscoring the importance of access to safe water, adequate sanitation, and hygiene for preventing the transmission of infectious diseases and protecting public health (1, 12). Furthermore, evidence indicates that the implementation of WASH programs is associated with reduced diarrheal disease, particularly among children in LMICs (11).

Schools provide a critical setting for WASH promotion because children spend a substantial proportion of their daily lives in school, where their health-related habits are established and reinforced, placing them at higher risk of contracting infectious diseases when schools have inadequate sanitation and a poor hygiene culture (13–15). Since access to clean water and sanitation facilities alone does not guarantee the protection of children's health, WASH services must also be functional, hygienic, accessible, and consistently maintained to prevent disease transmission effectively (16). Effective infection prevention and control (IPC) in school settings requires not only adequate WASH facilities but also appropriate knowledge, positive attitudes, and consistent hygiene practices to translate available resources into healthy behaviors (17). Consequently, integrating WASH infrastructure with health education and IPC measures can strengthen school WASH services, reduce the likelihood of disease transmission among students, instructors, and non-teaching staff, and support their regular school attendance (11, 17).

A growing body of literature has investigated WASH among school-aged children across different settings. A multi-country population study involving 75 LMICs assessed the prevalence of hygiene practices among adolescents aged 12 to 15 years (7). Moreover, in China, He et al. assessed KAP on sanitation and hygiene among primary school students (13), while Hao et al. specifically evaluated handwashing knowledge, attitudes, practices, and observed handwashing behaviors (18). Similarly, studies from Nepal and Nigeria evaluated WASH knowledge and practices, including comparisons between public and private school students (18, 19). In contrast, research from Guatemala primarily examined hygiene practices and factors associated with WASH behaviors (20), whereas studies in Japan and Nigeria focused on school WASH conditions and inequalities in WASH services (14, 21, 22). A recent study from India assessed and compared WASH-related knowledge, attitudes, and practices among adolescents attending government and non-government schools (23). Despite the growing body of evidence on school WASH, previous studies were conducted across diverse geographical and sociocultural settings and varied in their study populations, assessment methods, and outcome measures, limiting the generalizability of their findings to the Jordanian context.

Jordan, one of the most water-stressed countries in the world, has the second-lowest coverage of basic sanitary facilities in schools across Northern Africa and Western Asia, with only one in three Jordanian schools having basic sanitation facilities (24, 25). Despite having a lower mortality rate attributable to WASH-related diseases than the global average, Jordan continues to face an important public health burden from these diseases, particularly among children, as inadequate WASH remains a major contributor to diarrheal diseases (24, 26). In addition, the presence of cholera outbreaks in several neighboring countries highlights the potential risk of cross-border transmission, emphasizing the importance of strengthening WASH measures and disease prevention strategies in Jordan (27).

Despite these public health challenges, studies from Jordan have primarily focused on specific hygiene-related topics rather than WASH as a comprehensive concept. Earlier studies addressed handwashing habits among schoolchildren (28), while more recent research examined handwashing practices and associated barriers (29). Moreover, Qasass et al. assessed the menstrual KAP among adolescent schoolgirls (30), whereas Khamaiseh & Leimoon examined the personal hygiene knowledge, attitudes, and practices among primary school students (31). Although these studies have provided valuable insights into specific hygiene behaviors, they did not comprehensively assess knowledge, attitudes, and practices related to safe drinking water, sanitation, and hygiene within a single framework, nor did they compare WASH-related outcomes between students attending public and private schools.

Unlike previous Jordanian studies that focused on specific hygiene domains, this study provides the first comprehensive assessment of knowledge, attitudes, and practices related to safe drinking water, sanitation, and hygiene among pre-adolescent students, comparing public and private schools and thereby identifying priority areas for intervention and supporting evidence-based decision-making. The findings are expected to inform targeted school-based health promotion and health education programs to reduce disease exposure and strengthen infection prevention among Jordanian schoolchildren.

This study was guided by the Knowledge-Attitude-Practice (KAP) framework, which is widely used in public health research to examine health-related behaviors. The framework assumes that knowledge, attitudes, and practices are interrelated constructs and provides a useful basis for understanding hygiene-related behaviors. In addition, demographic and school-related characteristics may be associated with students' WASH knowledge, attitudes, and practices. Based on this framework, the present study examined the relationships among WASH knowledge, attitudes, practices, and selected demographic characteristics among Jordanian schoolchildren (32).

This study aimed to assess the level of WASH knowledge, attitudes, and practices among preadolescent students; to examine the relationships between students‘ WASH knowledge, attitudes, and practices and selected socio-demographics; to examine WASH-KAP differences between public and private schools; and to identify predictors of preadolescents' WASH practices.

Research Questions are as follows: 1. What level of knowledge, attitude, and practice related to WASH do school students in Jordan have? 2. Is there any association between WASH-KAP and socio-demographics? 3. Will the private school students show a higher level of KAP-related WASH? 4. “What are the predictors of preadolescent students' practices?”

## Methods

### Study design and setting

The study design is a descriptive, correlational, cross-sectional study that describes WASH-related knowledge, attitudes, and practices among preadolescent school students in Jordan. It investigates the correlations between students' WASH knowledge, attitudes, and practices and selected sociodemographic characteristics and compares KAP scores between public and private school students. The students were randomly selected from four private and four government schools in the Amman governorate.

The reference population in this study was public and private school students in Jordan. The accessible population of this study was preadolescent students who met the eligibility criteria. The inclusion criteria are as follows: (1) schools are located in Amman; (2) schools cater to education for students aged 9 to 12; and (3) schools are affiliated with the selected directorates. The exclusion criteria are (1) Schools outside the selected governorate and (2) Schools affiliated with non-selected directorates.

### Study sample and sampling method

The researchers selected a sample of 600 students from eight schools in Amman, including 300 from four public and 300 from four private schools. Each school type had an equal number of male and female participants, with 150 males and 150 females in the fifth, sixth, and seventh grades, ages 9–12.

A linear bivariate regression: one group, size of slope, was conducted using G^*^power 3.1 (33) to identify the required sample size with a one-tailed test and medium effect size *r* = 0.15, a statistical power of 0.80, and a significant alpha of 0.05. The desired sample size is 270. 12% was added to cover attrition; therefore, the minimum sample size should be 302. To enhance the representativeness of the study population and enable meaningful comparisons between public and private schools, a total of 600 participants were included (300 from public schools and 300 from private schools).

A Multistage stratified random sampling technique was applied to guarantee an equal selection of male and female participants from public and private schools. Stratified sampling designs subdivide the population into subsets from which elements are randomly selected. Stratification is often based on such demographic attributes as age or sex. By using stratified random sampling, researchers can sharpen the representativeness of their samples. There are 893 governmental schools and 1,439 private schools in Amman (27).

A four-stage stratified random sampling technique was employed. In the first stage, a list of all eligible schools was obtained from the Jordanian Ministry of Education, and four of the nine school directorates in Amman City were selected using a simple random sample technique to ensure geographic representation. In the second stage, schools were stratified by sector (public and private), and one public and one private school were randomly selected from each selected directorate. At the third stage, a student list was obtained from each school, and eligible students were stratified by sex and age group. At the final stage, students were randomly selected from each stratum to ensure equal representation of males and females.

### Questionnaire and psychometric properties

The data were collected through a self-administered questionnaire consisting of two parts; the first part was a sociodemographic questionnaire comprising 9 items regarding sociodemographic factors, including age, sex, type of school, place of residence, parents'/guardians' education, grade, household income, and number of household members. The second part was Knowledge, attitudes, and practices on water, sanitation, and hygiene, comprising three sections: Knowledge (18 items), Attitudes (24 items), and Practices (10 items). Responses were recorded as “yes” or “no” and scored dichotomously, with 1 assigned to a correct or favorable response and 0 to an incorrect or unfavorable response. Item scores were summed separately within each domain to obtain total knowledge, attitude, and practice scores. The possible score ranges were 0–18 for knowledge, 0–24 for attitudes, and 0–10 for practices, with higher scores indicating greater WASH knowledge, more favorable attitudes, and better WASH practices. A tertile-based classification approach was used to categorize scores into low, medium, and high levels based on the observed score distributions. Scores equal to or below the 33rd percentile (P33) were classified as low, scores greater than P33 and equal to or less than the 66th percentile (P66) as medium, and scores greater than P66 as high. The corresponding P33 and P66 values were 10 and 12 for knowledge, 15 and 19 for attitudes, and 5 and 7 for practices. Accordingly, knowledge scores were classified as low (≤ 10), medium (11, 12), and high (≥13); attitude scores as low (≤ 15), medium (16–19), and high (≥20); and practice scores as low (≤ 5), medium (6, 7), and high (≥8). The questionnaire was adapted from previously published studies (6, 16, 19), and translated into Arabic according to WHO translation guidelines. Minor linguistic and cultural adaptations were made to ensure the questionnaire was appropriate for the Jordanian context while preserving the items' original meaning. For example, terminology that was not commonly used in Jordan, such as “latrine,” was replaced with a more familiar equivalent. Furthermore, the researchers used a tool validated by WHO and UNICEF, including 17 core questions and indicators to observe and record school characteristics related to WASH (12).

The questionnaire was reviewed by a panel of five experts in public health and school health to assess its content validity and cultural appropriateness. The questionnaire was validated and met a satisfactory level; the S-CVI average based on proportional relevance was 0.95, and the S-CVI Universal agreement average was 0.87, indicating that the survey questionnaire is valid.

The questionnaire's feasibility and reliability were tested before data collection; the researchers conducted a pilot study with 16 students. The internal consistency of Cronbach's alpha of 0.705 indicates that the questionnaire is reliable.

### Data collection procedure

The data collection procedure was conducted over 3 months (September, October, and November 2024). Researchers visited the schools, informed the directors of the study objectives and procedures, obtained permission to collect data, and distributed parental/guardian consent forms. They then identified participants who met the eligibility criteria and agreed to participate in the study. Participants who met the inclusion criteria and voluntarily returned the consent form to participate in the survey provided a sufficient explanation of the study's objectives and potential benefits. A self-report questionnaire was distributed to the students to complete after reading it with the researchers, with assurances of anonymity. The questionnaires were returned to the researchers and kept in a secure file devoted by the researchers for each school. For each school, the researchers completed the characteristics report based on direct observation of the toilet and drinking water facilities, with minimal assistance from the school director.

### Ethical consideration

The study's ethical approval was granted by the Jordanian Ministry of Education and the schools‘ directorates (protocol code 42074/10/3; date of approval: 2/9/2024). A consent form was signed by each subject's parent or guardian before the survey, containing a thorough description of the study's purpose and clearly explaining that participation is voluntary and that subjects' anonymity is preserved.

### Statistical analysis

The Statistical Package for the Social Sciences (SPSS) version 26 was used for analysis based on the research questions. It is necessary to check the data and set errors prior analysis, this stage is known as the pre-analysis phase and involves the screening and cleaning of the data. Also, include checks of the assumptions of Pearson correlation, independent *t*-test, and regression; this stage identifies problems in the data before analysis to ensure proper analysis. No missing data were found.

Descriptive statistics (mean, percentage, and standard deviation) were used to describe the study sample demographics. Other statistical tests, including the independent *t*-test and Pearson correlation, were used to examine differences in mean scores for knowledge, attitude, and practices by students' demographic variables. A hierarchical multiple regression analysis was used to identify the predictors of practices.

## Results

### School characteristics

The WASH characteristics of the participating schools are presented in Table 1. Overall, differences were observed between public and private schools in drinking water sources, toilet types, availability of soap and water, and the number of staff and student toilets.

**Table 1:** School characteristics.

Questions		Public *n* (%)	Private *n* (%)	Overall *n* (%)
Categorical variables				
Main drinking water provided at schools	Pipped water supply	3 (37.5%)	1 (12.5%)	4 (50%)
	Packaged bottled water	0 (0%)	3 (37.5%)	3 (37.5%)
	No water source	1 (12.5%)	0 (0%)	1 (12.5%)
Type of toilets	flush toilets	0 (0%)	4 (50%)	4 (50%)
	Pour-flush toilets	4 (50%)	0 (0%)	4 (50%)
Soap and water availability	Water and soap	0 (0%)	4 (50%)	4 (50%)
	water only	4 (50%)	0 (0%)	4 (50%)
Water treatment/purification methods		0 (0%)	4 (50%)	4 (50%)
Water quality test		2 (25%)	1 (12.5%)	3 (37.5%)
Water in classrooms		0 (0%)	0 (0%)	0 (0%)
WASH education		0 (0%)	2 (25%)	2 (25%)
handwashing facilities at the school		4 (50%)	4 (50%)	8 (100%)
Separated toilet facilities for girls and boys		2 (25%)	4 (50%)	6 (75%)
Hand wash practice education		1 (12.5%)	−12.50%	2 (25%)
Water is always available in the toilets.		2 (25%)	4 (50%)	6 (75%)
Soap is provided out the toilets for washing hands.		0 (0%)	0 (0%)	0 (0%)
Clean toilet facilities		0 (0%)	4 (50%)	4 (50%)
Drinking water is available on each floor of the school.		0 (0%)	4 (50%)	4 (50%)
		**Mean**	**SD**	
Continuous variables				
Staff toilet's total number	Public	1.00	0.000	
	Private	2.25	0.500	
Student's total toilet number	Public	5.75	2.062	
	Private	10.50	2.517	

### Demographic characteristics of study participants

Seven hundred questionnaires were distributed, and 600 were completed, with a good response rate of 85%. Table 2 summarizes the demographic characteristics of the study participants by school type. The distribution of age, sex, and grade was comparable between public and private schools. In contrast, significant differences were observed in household size, monthly household income, and parents' educational levels (*p* < 0.001).

**Table 2:** Demographic characteristics of study participants.

Characteristics	Private *n* (%)	Public *n* (%)	Total *n* (%)	*p*-value
**Sex**				**1.000**
Male	150 (50.0)	150 (50.0)	300 (50.0)	
Female	150 (50.0)	150 (50.0)	300 (50.0)	
**Grade**				**1.000**
5th grade	100 (33.3)	100 (33.3)	200 (33.3)	
6th grade	100 (33.3)	100 (33.3)	200 (33.3)	
7th grade	100 (33.3)	100 (33.3)	200 (33.3)	
**Monthly household income**				**<0.001**
<500 JD	19 (6.3)	127 (42.3)	146 (24.3)	
500–1000 JD	140 (46.7)	124 (41.3)	264 (44.0)	
>1000 JD	141 (47.0)	49 (16.3)	190 (31.7)	
**Mother's education**				**<0.001**
High school or less	70 (23.3)	141 (47.0)	211 (35.2)	
University degree	230 (76.7)	159 (53.0)	389 (64.8)	
**Father's education**				**<0.001**
High school or less	67 (22.3)	142 (47.3)	209 (34.8)	
University degree	233 (77.7)	158 (52.7)	391 (65.2)	
**Age (years), mean** **±SD**	10.80 ± 0.97	10.86 ± 0.95	10.83 ± 0.96	**0.394**
**Household size, mean** **±SD**	5.39 ± 1.34	6.25 ± 1.57	5.82 ± 1.52	**<0.001**

Bold formatting is used to distinguish the main characteristic categories and the corresponding *p*-values.

### Knowledge, attitudes, and practices scores

Table 3 presents the descriptive statistics and distribution of participants across the KAP levels. Based on the tertile-based classification described in the Methods, the largest proportion of participants had low WASH knowledge (43.8%), whereas moderate levels were most frequently observed for attitudes (39.8%) and practices (36.2%).

**Table 3:** Descriptive statistics and comparison of knowledge, attitude, and practice scores by school type.

KAP Domains	Overall Mean ±SD	Min-Max	P33	P66	Public Mean ±SD	Private Mean ±SD	*t*	df	*p*-value
Knowledge	10.46 ± 2.62	2–14	10	12	10.31 ± 2.75	10.61 ± 2.47	1.401	598	0.161
Attitude	16.27 ± 3.78	4–23	15	19	15.14 ± 3.91	17.38 ± 3.29	7.759	598	<0.001
Practices	6.20 ± 1.98	0–9	5	7	5.65 ± 2.04	6.73 ± 1.74	6.970	598	<0.001

### Inferential statistics

#### Correlations among the study variables

Pearson's correlation analysis showed significant positive associations among WASH knowledge, attitudes, and practices. Knowledge was positively correlated with both attitudes and practices, and attitudes were positively correlated with practices (all *p* < 0.001). Age and grade were also positively associated with KAP scores, while sex was significantly associated with practices, with female students demonstrating better practice scores (Table 4).

**Table 4:** Correlations among the study variables.

Variable	Practices	Attitude	Knowledge	Age	Family #	Grade	Sex
Practices	1						
Attitude	0.561**	1					
Knowledge	0.392**	0.580**	1				
Age	0.145**	0.189**	0.217**	1			
Family #	0.062	0.098*	0.028	0.023	1		
Grade	0.128**	0.215**	0.228**	0.918**	0.007	1	
Sex	0.190**	0.000	0.11	0.000	0.051	0.064	1

^**^Correlation is significant at the 0.01 level (2-tailed).

^*^Correlation is significant at the 0.05 level (2-tailed).

#Indicates family size (number of household members).

#### Comparison of knowledge, attitude, and practice scores between public and private school students

Independent-samples *t*-tests showed that attitude and practice scores were significantly higher among private school students, whereas knowledge scores did not differ significantly between public and private school students (Table 3). Despite the absence of a significant difference in overall knowledge scores, significant differences were observed for several individual knowledge items, particularly those related to the use of soap for handwashing, receipt of WASH information during the previous 6 months, water quality, hand hygiene, and the role of human feces in transmitting infection. Detailed item-level results are presented in Supplementary Table S1.

### Predictors of students practices

Hierarchical linear regression showed that the proportion of variance in WASH practices explained by the model increased progressively after adding knowledge and attitudes, with the final model explaining 36.4% of the variance (Table 5). In the final model, attitudes were the strongest positive predictor of WASH practices, followed by sex, school type, and knowledge, whereas age and household size were not significant predictors.

**Table 5:** Predictors of students practices.

Variables	Step 1 β (*p*-value)	Step 2 β (*p*-value)	Step 3 β (*p*-value)
Sex	0.190 (<0.001)	0.172 (<0.001)	0.156 (<0.001)
School type	0.284 (<0.001)	0.256 (<0.001)	0.145 (<0.001)
Age	0.154 (<0.001)	0.077 (0.034)	0.042 (0.212)
Family #	0.014 (0.730)	−0.002 (0.959)	0.016 (0.647)
Knowledge	—	0.352 (<0.001)	0.114 (0.006)
Attitudes	—	—	0.436 (<0.001)
R^2^	0.135	0.252	0.364
ΔR^2^	—	0.117	0.112
Model fit	F = 23.267, *p* < 0.001	F = 93.002, *p* < 0.001	F = 103.999, *p* < 0.001
Variables	Step 1 β (*p*-value)	Step 2 β (*p*-value)	Step 3 β (*p*-value)

#Indicates family size (number of household members).

## Discussion

This study revealed important deficiencies in essential WASH services in public schools in Jordan. This is not just a concern but an alarming situation that demands immediate attention. Even when schools had WASH infrastructure, several essential components, including WASH education, soap, functional flush toilets, and sufficient neat toilet facilities to attend to and protect the school community's health, were absent in most public schools. Furthermore, drinking water was not consistently available on every floor of the schools, and most of the schools relied on a piped water supply without any purification as their primary source of drinking water. These deficiencies may limit students' ability to translate their knowledge into appropriate hygiene practices, and increase their vulnerability to hygiene-related diseases. Our findings align with a body of literature that highlights the lack of school facilities, which may negatively affect students' hygiene practices, and the importance of adequate WASH facilities in promoting healthy behaviors. Several findings have been reported in previous studies conducted in LMICs, where the WASH infrastructure was identified as a major barrier among schoolchildren to maintain appropriate hygiene behaviors (13, 14, 18, 29). Collectively, these findings reinforce the importance of providing adequate WASH infrastructure alongside hygiene education to achieve sustainable improvements in students' health and hygiene behaviors.

Assessing schoolchildren's Knowledge, Attitudes, and Practices regarding WASH provides valuable insights into the factors influencing hygiene behaviors in School settings. In our study, noteworthy pre-adolescent students demonstrated low levels of knowledge and moderate attitudes and WASH practices. Similar findings have been reported in studies conducted in Limpopo, India, Nepal, Rwanda, Botswana, and the Philippines, where gaps between WASH knowledge and actual hygiene practices were also observed (16, 19, 34–37). These findings highlight the need for comprehensive school-based health education and behavioral interventions that not only strengthen WASH knowledge but also translate knowledge and positive attitudes into appropriate hygiene practices, thereby promoting children's health and preventing WASH-related diseases. In contrast, a study conducted among Japanese elementary school students reported consistently high levels of WASH knowledge, attitudes, and practices. This difference may reflect variations in school health policies, the availability and quality of clean and functional WASH facilities, continuous access to soap and safe drinking water, and the integration of WASH education into the national curriculum (22).

Consistent with the findings of He et al. from a Chinese study (13), the present study revealed that higher WASH knowledge translated into better attitudes and practices among Jordanian school students, supporting the role of knowledge as an important determinant of healthy behaviors. In contrast, Hothur et al. reported no significant relationship between Indian students' knowledge and practices (38). discrepancy may be attributed to differences in the school environment, the availability of WASH facilities, hygiene promotion strategies, or cultural and contextual factors that influence the translation of knowledge into practices. This also suggests that knowledge alone may not be sufficient to improve hygiene practices unless students have access to an enabling environment that supports the adoption of appropriate hygiene and sanitation behaviors.

The present study further demonstrated that irrespective of sex, older students and those in higher grades had better WASH knowledge, attitudes, and practice. This result concurs with previous studies conducted in Jordan, Namibia, and India (2015, 2018, and 2021, respectively), which reported that students become more independent and more aware of sanitation and hygiene precautions as they age (39–42). This may be explained by the cumulative effect of school-based health education efforts and gradual development of students' cognitive and behavioral skills, which enable older students to better understand and implement appropriate WASH practices.

Aligned with previous studies, the present study found no evident associations between sex and WASH knowledge and attitudes (40, 43). However, female students demonstrated better WASH practices than male students, which is consistent with Wada O et al. findings (14). This difference may be attributed to increased adherence to personal hygiene practices, higher levels of health awareness, and increased parental and social support for hygiene behaviors among girls.

Additionally, our findings suggest that there were no significant differences in overall WASH knowledge between students attending private and public schools, albeit private school students demonstrated significantly better knowledge in several individual WASH-related items, including the importance of using hand soap, awareness that human feces contain germs that can cause infection, and receiving WASH education in the last 6 months. Furthermore, private school students exhibited significantly better WASH attitudes and practices than their counterparts in public schools. These findings indicate disparities in WASH knowledge, attitudes, and practices between public and private schools and suggest that, although the overall knowledge scores were comparable, differences in the school environment, including the availability of WASH facilities, hygiene resources, and school-based WASH education, may have contributed to the more positive attitudes and better hygiene practices observed among private school students. Similar findings have been reported in previous studies, which demonstrated that private school students had better WASH awareness, attitudes, and practices than public school students. Patel et al. reported that private school students had better WASH knowledge, attitudes, and practices than those in public schools (44). Likewise, Bosede et al. reported comparable findings, although a slight difference was observed regarding WASH knowledge, which was higher among public school students (18). In contrast, Ommy Mushota et al. reported poorer WASH performance among Indian private school students compared to students attending public school (41). These contrasting findings may reflect differences in school resources, water, sanitation, hygiene infrastructure, educational programs, and socioeconomic and contextual characteristics across the study settings.

The hierarchical linear regression analysis showed that students' demographic factors, including sex, school type, and age, were significant predictors of students' practices. Females, students in private schools, and older students were more likely to report better WASH practices than male students and students attending public schools; these findings align with the body of literature indicating that school type is a significant predictor of hygienic practices (14, 45, 46). Others reported that male and younger students were significantly associated with lower levels of practices (8, 13). Contrary to our findings, a study from Guatemala found that better school type negatively correlated to good hygiene practices (20). Moreover Patel et al. (44) confirmed the absence of a relation between age and students' practices. Variations in the educational and environmental contexts across studies may explain these findings.

Furthermore, attitudes were a significant predictor for practices in the second model after controlling for all demographic characteristics. In the final model, controlling for demographics and knowledge, attitudes remained a significant predictor of practices, indicating that the higher the level of student knowledge and positive attitudes were associated with better WASH practices. Similar findings have been reported in Studies from different parts of the world, which also found positive associations between positive hygiene practices and higher knowledge levels (8, 15, 47). This association may reflect that students with greater knowledge of WASH and more positive attitudes are more aware of recommended hygiene behaviors and, therefore, more likely to report appropriate WASH practices.

Overall, the study findings have significant educational and public health implications. The observed differences in WASH knowledge, attitudes, and practices, together with the deficiencies identified in school WASH services, underscore the need to strengthen comprehensive school-based WASH programs in Jordan, particularly in public schools. Efforts should focus on integrating WASH education with improvements in school infrastructure, including ensuring continuous access to safe drinking water, soap, and adequate sanitation facilities. Such measures may support healthier hygiene behaviors, reduce the risk of WASH-related diseases, and contribute to creating healthier and safer learning environments for schoolchildren.

This study is the first in Jordan and the surrounding regions to assess school students' WASH knowledge, attitudes, and practices among students aged 9 to 12 years old. The strengths of our study were the large sample size and the multistage stratified random sampling technique study method that guaranteed an equal selection of male and female participants, which sharpened the sample's representativeness. However, it had a few limitations, including the self-reported questionnaire, as respondents may overestimate or underestimate their responses, and the cross-sectional design; this study design does not allow the researcher to infer the cause or consequences of differences between or among groups of participants. Another limitation of this study is that household income was self-reported by children aged 9–12 years. Although simplified income categories were used to minimize reporting difficulty, children in this age group may not have precise knowledge of their monthly household's income. Therefore, income data may reflect perceived socio-economic status rather than exact monthly household income.

Future research should focus on alternative methodologies with experimental design to enhance the behaviors of disadvantaged demographic students. Schools and governments play an essential and integral role in creating and supporting the physical environment by providing sufficient and clean toilet facilities with soap, proper flush toilets, and purified drinking water distributed on each school floor and designing awareness and education campaigns for students that aim to reduce the incidence of WASH-related diseases, enhance school enrollment, performance, and attendance, and enable children to act as agents of change in their households and communities.

Policymakers should develop and enforce minimum standards on WASH for all schools aligned with the World Health Organization and UNICEF, including periodically safe drinking water access, functional and clean handwashing facilities provided with hygiene supplies, and minimizing inequalities between public and private schools. Moreover, a dedicated budget for school WASH should be allocated to fund infrastructure construction and maintenance in schools and implement continuous monitoring and accountability systems.

## Conclusion

This study provided essential insights into WASH-related knowledge, attitudes, and practices among preadolescent students in public and private schools in Amman, Jordan, and identified significant gaps in WASH education, soap availability, access to clean, flush toilets, and drinking water facilities in public schools, while also providing a roadmap for targeted improvements. Key findings were that students in public schools demonstrated significantly poor WASH-related attitudes, practices, and specific knowledge compared to private schools. However, the study also revealed that older age and higher grades were associated with superior WASH-related knowledge, attitudes, and practices outcomes, indicating a potential for improvement. Furthermore, sex, school type, age, knowledge, and attitudes were significant predictors of students' practices. These results underscore the need for intervention to fill gaps and address the challenges.

## Data Availability

The raw data supporting the conclusions of this article will be made available by the authors, without undue reservation.

## References

[1] UN-Water. The United Nations World Water Development Report 2021: Valuing Water. Paris. (2021). Available online at: https://www.unwater.org/publications/un-world-water-development-report-2021 [Accessed August 22, 2025]

[2] Water: A Shared Responsibility – The United Nations World Water Development Report 2. Dev Pract. (2007) 17:309–311. doi: 10.1080/09614520701197333

[3] Farah S Karim M Akther N Begum M Begum N. Knowledge and Practice of Personal Hygiene and Sanitation: A Study in Selected Slums of Dhaka City. Delta Med Coll J. (2015) 3:68–73. doi: 10.3329/dmcj.v3i2.24425

[4] Mohd R Malik I. Sanitation and Hygiene Knowledge, Attitude and Practices in Urban Setting of Bangalore: A Cross-Sectional Study. J Community Med Health Educ. (2017) 7:540. doi: 10.4172/2161-0711.1000540

[5] Sridhar MKC Okareh OT Mustapha M. Assessment of Knowledge, Attitudes, and Practices on Water, Sanitation, and Hygiene in Some Selected LGAs in Kaduna State, Northwestern Nigeria. J Environ Public Health. (2020) 2020:1–14. doi: 10.1155/2020/6532512 32934659 PMC7479483

[6] Berhe AA Aregay AD Abreha AA Aregay AB Gebretsadik AW Negash DZ . Knowledge, Attitude, and Practices on Water, Sanitation, and Hygiene among Rural Residents in Tigray Region, Northern Ethiopia. J Environ Public Health. (2020) 2020:1–9. doi: 10.1155/2020/5460168 32256616 PMC7106921

[7] Han L Gao X Liao M Yu X Zhang R Liu S . Hygiene practices among young adolescents aged 12-15 years in low- and middle-income countries: a population-based study. J Glob Health. (2020) 10:020436. doi: 10.7189/jogh.10.020436 33312503 PMC7719273

[8] Hsan K Naher S Griffiths MD Shamol HH Rahman MA. Factors associated with the practice of water, sanitation, and hygiene (WASH) among the Rohingya refugees in Bangladesh. J Water Sanit Hyg Dev. (2019) 9:794–800. doi: 10.2166/washdev.2019.038

[9] UNESCO. Imminent risk of a global water crisis, warns the UN World Water Development Report. (2023) Available online at: https://www.unesco.org/en/articles/imminent-risk-global-water-crisis-warns-un-world-water-development-report-2023 [Accessed August 22, 2025]

[10] United Nations Environment Programme. Globally, 3 billion people at health risk due to scarce data on water quality. (2021) Available online at: https://www.unep.org/news-and-stories/story/globally-3-billion-people-health-risk-due-scarce-data-water-quality [Accessed January 30, 2025]

[11] Prüss-Ustün A Wolf J Bartram J Clasen T Cumming O Freeman MC . Burden of disease from inadequate water, sanitation and hygiene for selected adverse health outcomes: An updated analysis with a focus on low- and middle-income countries. Int J Hyg Environ Health. (2019) 222:765–77. doi: 10.1016/j.ijheh.2019.05.004 31088724 PMC6593152

[12] World Health Organization; UNICEF. Core questions and indicators for monitoring WASH in Schools in the Sustainable Development Goals. (2016).

[13] He J Zeng Y Hao M Yamauchi T. Knowledge, Attitudes and Practices of Sanitation and Hygiene among Primary School Students in Rural Area of Northeast China. Sanitation Value Chain. (2020) 4:39–050.

[14] Wada OZ Olawade DB Oladeji EO Amusa AO Oloruntoba EO. School water, sanitation, and hygiene inequalities: a bane of sustainable development goal six in Nigeria. Can J Public Health. (2022) 113:622–35. doi: 10.17269/s41997-022-00633-9 35411423 PMC8999996

[15] Assefa M Kumie A. Assessment of factors influencing hygiene behaviour among school children in Mereb-Leke District, Northern Ethiopia: a cross-sectional study. BMC Public Health. (2014) 14:1000. doi: 10.1186/1471-2458-14-1000 25256291 PMC4190334

[16] Vishnupriya S Prasad S Kasav JB Trout K Murthy S Surapaneni KM . Water and sanitation hygiene knowledge, attitudes and practices among school settings in rural Chennai. J Water Sanit Hyg Dev. (2015) 5:192–200. doi: 10.2166/washdev.2014.052

[17] UNICEF. WASH and infection prevention and control measures in schools. (2020) Available online at: https://www.unicef.org/documents/wash-and-infection-prevention-and-control-measures-schools [Accessed January 30, 2025]

[18] Bosede AO Enwenonu RC Udensi JU Ihejirika OC Akindulureni KC Emenonu OC . Comparative assessment of WASH adherence among public and private school students in a rural district in Nigeria. BMC Public Health. (2025) 25:2014. doi: 10.1186/s12889-025-23253-7 40450261 PMC12125929

[19] Shrestha MV Manandhar N Joshi SK. Study on Knowledge and Practices of Water, Sanitation and Hygiene among Secondary School Students. J Coll Med Sci Nepal. (2018) 14:160–5. doi: 10.3126/jcmsn.v14i3.21158

[20] Pieters MM Fahsen N Quezada R Pratt C Craig C McDavid K . Assessing hand hygiene knowledge, attitudes, and behaviors among Guatemalan primary school students in the context of the COVID-19 pandemic. BMC Public Health. (2023) 23:2252. doi: 10.1186/s12889-023-17168-4 37974121 PMC10652458

[21] Wada OZ Oloruntoba EO. Safe Reopening of Schools during COVID-19: An Evaluation of Handwash Facilities and Students' Hand Hygiene Knowledge and Practices. Eur J Environ Public Health. (2021) 5:em0072. doi: 10.21601/ejeph/9704

[22] Sugita EW. Water, Sanitation and Hygiene (WASH) in Japanese elementary schools: Current conditions and practices. Pediatr Int. (2022) 64:e15062. doi: 10.1111/ped.15062 34787938 PMC9313865

[23] Patel M Gupta EK Yogesh M. Assessing and comparing knowledge, attitude, and practices related to water, sanitation and hygiene among government and non-government school students in Gujarat: a mixed method study. BMC Public Health. (2025) 25:1768. doi: 10.1186/s12889-025-22937-4 40369474 PMC12077077

[24] UNICEF. Tapped out: The costs of water stress in Jordan. (2022). Available online at: https://www.unicef.org/jordan/media/11356/file/water%20stress%20in%20Jordan%20report.pdf [Accessed August 22, 2025]

[25] UNICEF. Drinking Water, Sanitation and Hygiene in Schools: Global Baseline Report 2018. (2018). Available online at: https://www.unicef.org/reports/drinking-water-sanitation-and-hygiene-schools [Accessed January 30, 2025]

[26] UNICEF. Diarrhoeal disease. (2024) Available online at: https://data.unicef.org/topic/child-health/diarrhoeal-disease [Accessed August 12, 2026]

[27] Ministry of Education. Annual Statistical Report for the Academic Year 2022/2023. (2023). Available online at: https://moe.gov.jo/sites/default/files/tqryr_hsyy_2022-2023_nhyy.pdf [Accessed August 12, 2026]

[28] ALBashtawy M. Assessment of hand-washing habits among school students aged 6–18 years in Jordan. Br J Sch Nurs. (2017) 12:30–6. doi: 10.12968/bjsn.2017.12.1.30

[29] Abu Aqab A. Hand-washing Practice, Barriers, and Associated Factors among Jordanian Students in Public and Private Schools: A Cross-sectional Study. J Jordan Nurs Res. (2026) 5:2. doi: 10.14525/JJNR.2026.13

[30] Qasass Y Albashtawy M Mohammad KI Hamaideh SH. Ta'an W, Rayan A, Hamadneh J, Hamadneh S, Al-Awamreh K, Ayed A, et al. Knowledge and Practices of Menstrual Hygiene among Adolescent Schoolgirls in Jordanian Badia Region: A Field Study. J Jordan Nurs Res. (2023) 2:219–30.

[31] Khamaiseh A Leimoon H. Knowledge, attitude, and practice of personal hygiene among primary school students in southern Jordan: A cross-sectional school-based study. (2024) doi: 10.21203/rs.3.rs-3849230/v1

[32] Vargas-Garrido H. Defining conceptual boundaries of knowledge attitudes and practices to improve validity in public health research. Discover Public Health. (2026) 23:978. doi: 10.1186/s12982-026-02372-5

[33] Faul F Erdfelder E Buchner A. Lang A-G. Statistical power analyses using G^*^Power 31: Tests for correlation and regression analyses. Behav Res Methods. (2009) 41:1149–60. doi: 10.3758/BRM.41.4.1149 19897823

[34] Sibiya J Gumbo J. Knowledge, Attitude and Practices (KAP) Survey on Water, Sanitation and Hygiene in Selected Schools in Vhembe District, Limpopo, South Africa. Int J Environ Res Public Health. (2013) 10:2282–95. doi: 10.3390/ijerph10062282 23736657 PMC3717737

[35] Thakadu OT Ngwenya BN Phaladze NA Bolaane B. Sanitation and hygiene practices among primary school learners in Ngamiland district, Botswana. Phys Chem Earth Parts A/B/C. (2018) 105:224–30. doi: 10.1016/j.pce.2018.02.006

[36] Sangalang SO Medina SAJ Ottong ZJ Lemence ALG Totanes D Valencia JC . Protocol for a Trial Assessing the Impacts of School-Based WaSH Interventions on Children's Health Literacy, Handwashing, and Nutrition Status in Low- and Middle-Income Countries. Int J Environ Res Public Health. (2020) 18:226. doi: 10.3390/ijerph18010226 33561075 PMC7795080

[37] Mourad KA Habumugisha V Sule BF. Assessing Students' Knowledge on WASH-Related Diseases. Int J Environ Res Public Health. (2019) 16:2052. doi: 10.3390/ijerph16112052 31185642 PMC6604011

[38] Hothur R Arepalli S Doddoju Veera Bhadreshwara A. A KAP study on water, sanitation and hygiene among residents of Parla village, Kurnool district, Andhra Pradesh. Int J Community Med Public Health. (2019) 6:2081. doi: 10.18203/2394-6040.ijcmph20191823

[39] ALBashtawy M. Personal hygiene in school children aged 6–12 years in Jordan. Br J Sch Nurs. (2015) 10:395–8. doi: 10.12968/bjsn.2015.10.8.395

[40] Shilunga APK Amukugo HJ Mitonga KH. Knowledge, attitudes and practices of primary schools learners on sanitation and hygiene practices. Int J Community Med Public Health. (2018) 5:3197. doi: 10.18203/2394-6040.ijcmph20183051

[41] Mushota O Mathur A Pathak A. Effect of School-Based Educational Water, Sanitation, and Hygiene Intervention on Students' Knowledge in a Resource-Constrained Setting (2020). doi: 10.21203/rs.3.rs-122523/v1 PMC8666030 34895193

[42] Mushota O Mathur A Pathak A. Effect of school-based educational water, sanitation, and hygiene intervention on student's knowledge in a resource-limited setting. BMC Public Health. (2021) 21:2258. doi: 10.1186/s12889-021-12279-2 34895193 PMC8666030

[43] Giardina D Prandini F Sorlini S. Integrated Assessment of the Water, Sanitation and Hygiene Situation in Haitian Schools in the Time of Emergency. Sustainability. (2013) 5:3702–21. doi: 10.3390/su5093702

[44] Patel M Gupta EK Yogesh M. Assessing and comparing Knowledge, Attitude, and Practices related to Water, Sanitation and Hygiene Among Government and Non-government School students in Gujarat: A Mixed-Method Study. (2024) doi: 10.21203/rs.3.rs-3825718/v1 PMC12077077 40369474

[45] Admasie A Guluma A Feleke FW. Handwashing Practices and Its Predictors Among Primary School Children in Damote Woide District, South Ethiopia: An Institution Based Cross-Sectional Study. Environ Health Insights. (2022) 16:11786302221086795. doi: 10.1177/11786302221086795 35321321 PMC8935579

[46] Mane AB Reddy NS Reddy P KV C Nair SS T S. Differences of Hand Hygiene and its Correlates among School going Children in Rural and Urban Area of Karnataka, India. Arch Med. (2016) 8:5. doi: 10.21767/1989-5216.1000163

[47] Essa MSI Yousif MEA Abdalmagid MA. Role of Health Education in Promoting Knowledge, to-wards Personal Hygiene Among Primary School Pupils in Umbada locality, Khartoum State, Sudan. (2018 - 2020). ABC Research Alert. (2022) 10:45–55. doi: 10.18034/ra.v10i3.631

