# Supplementary material for: Knowledge, attitudes, and practices of safe drinking water, sanitation, and hygiene among pre-adolescent students in public and private schools in Jordan: cross-sectional school-based study
Source: Front Public Health. 2026 Sep 8;14:1925764. doi: 10.3389/fpubh.2026.1925764 (PMC13593898; doi:10.3389/fpubh.2026.1925764)
Supplement: Supplementary file 1 [file Table_1.docx]

**Knowledge, Attitudes, and Practices of Safe Drinking Water, Sanitation, and Hygiene Questionnaire for School Students**

**Section 1: Sociodemographic**

Before you begin, we ask that you answer some of the following personal questions by circling the correct answer or filling in the blank completely.

| 1. What is your sex? | A. Male B. Female |
| --- | --- |
| 2. What type of school do you attend? | A. Public B. Private |
| 3. Place of residence | A. Amman Governorate B. Another Governorate |
| 4. How old are you? | _______ years |
| 5. What grade are you in? | A. Grade 5 B. Grade 6   C. Grade 7 D. Grade 8  E. Grade 9 F. Grade 10 |
| 6. What is the number of your household members? | ________ members |
| 7. What is the highest degree or level of education your mother has completed? | A. Primary School. B. Secondary school.  C. High School D. Bachelor's Degree  E. Master's Degree F. Ph.D. or higher |
| 8. What is the highest degree or level of education your father has completed? | A. Primary School. B. Secondary school.  C. High School D. Bachelor's Degree  E. Master's Degree F. Ph.D. or higher |
| 9. What is your family's monthly household income? | A. Less than 500 Jordanian Dinars B. Between 500 and 1,000 Jordanian Dinars C. More than 1,000 Jordanian Dinars |

**Section 2: Knowledge on WASH**

Please answer the following questions based on your **knowledge** of water, sanitation, and hygiene (WASH) by circling either "Yes" or "No" for each statement.

| 1. Can unsafe water cause diarrheal diseases? | Yes | NO |
| --- | --- | --- |
| 2. Can water get contaminated? | Yes | NO |
| 3. Should a clean water source be used for hand washing? | Yes | NO |
| 4. Is washing hands with soap the same as washing them without soap? | Yes | NO |
| 5. Do the consequences of not washing hands expose to various diseases? | Yes | NO |
| 6. Do liquid wastes (sewage) expose to disease? | Yes | NO |
| 7. Does animal dung cause diseases? | Yes | NO |
| 8. Is drinking clean water considered a prevention mechanism for acute watery diarrhea? | Yes | NO |
| 9. Sharing drinking cups without washing brings health problems? | Yes | NO |
| 10. Is hand hygiene considered a prevention mechanism for acute watery diarrhea? | Yes | NO |
| 11. Using a toilet and Proper Sanitation, consider a prevention mechanism for acute watery diarrhea? | Yes | NO |
| 12. Are toilets necessary and required in every school? | Yes | NO |
| 13. Have you got information on WASH in the last 6 months? | Yes | NO |
| 14. Was the information received on WASH about Water quality? | Yes | NO |
| 15. Was the information received on WASH about toilets and sanitation? | Yes | NO |
| 16. Was the information received on WASH about Hand hygiene? | Yes | NO |
| 17. Human feces contain germs that can cause infection | Yes | NO |
| 18. Washing hands after a meal is more important than doing it before a meal | Yes | NO |

**Section 3: Attitude on WASH**

Please answer the following questions based on your **attitudes and beliefs** toward water, sanitation, and hygiene (WASH) by circling either "Yes" or "No" for each statement.

| 1. Do you believe that clean water consumption is important at all times, not only when one gets sick? | Yes | NO |
| --- | --- | --- |
| 2. Do you believe that consuming safe and enough water can prevent waterborne diseases? | Yes | NO |
| 3. Do you believe that defecating near a water source can cause contamination? | Yes | NO |
| 4. Do you believe that boiling water before consumption helps remove disease-causing microorganisms? | Yes | NO |
| 5. Do you believe that water containers must always be clean? | Yes | NO |
| 6. Do you believe that cattle dung, if not properly managed, causes health problems? | Yes | NO |
| 7. Do you believe that diarrheal diseases are caused by poor personal hygiene and sanitation? | Yes | NO |
| 8. Do you believe that diarrheal diseases are transmittable? | Yes | NO |
| 9. Do you believe that waste can be a breeding site for flies and rodents? | Yes | NO |
| 10. Do you believe that every school has an obligation to construct enough latrines for students and staff? | Yes | NO |
| 11. Do you believe that latrine structures should include ventilation? | Yes | NO |
| 12. Do you believe that latrines should be cleaned daily? | Yes | NO |
| 13. Do you believe that washing hands after using the latrine prevents diarrheal diseases? | Yes | NO |
| 14. Do you believe that children’s stool contains disease-causing germs? | Yes | NO |
| 15. Do you believe that washing hands requires soap, not just water alone, to sanitize hands? | Yes | NO |
| 16. Do you believe that washing hands is important both before and after eating food? | Yes | NO |
| 17. Do you believe that washing hands is important to prevent infections and remove germs, not just for feeling clean? | Yes | NO |
| 18. Do you believe that a mother’s unhygienic hands can cause health problems? | Yes | NO |
| 19. Do you believe that hand hygiene is related to diarrheal diseases? | Yes | NO |
| 20. Do you believe that bathing is important for health, not just for beauty? | Yes | NO |
| 21. Do you believe that disposing of liquid waste inside the compound can cause health problems? | Yes | NO |
| 22. Do you believe that the significance of a latrine goes beyond privacy, serving important health purposes? | Yes | NO |
| 23. Do you believe that non-utilization of a latrine by some students can affect the health of other students? | Yes | NO |
| 24. Do you believe that latrine use is important at all times, not only at night? | Yes | NO |

**Section 4: Practice of water and sanitation hygiene**

Please answer the following questions based on your water, sanitation, and hygiene (WASH) **practices** by circling either "Yes" or "No" for each statement**.**

| 1. Do you drink water in your school directly from the tap? | Yes | NO |
| --- | --- | --- |
| 2. Do you bring water from home to drink? | Yes | NO |
| 3. Do you usually wash your hands before a meal? | Yes | NO |
| 4. Do you usually wash your hands after a meal? | Yes | NO |
| 5. Do you usually wash your hands after defecation? | Yes | NO |
| 6. Do you regularly clean your teeth (twice at least)? | Yes | NO |
| 7. Do you wash your feet every day? | Yes | NO |
| 8. Do you always wash your hands with water and soap? | Yes | NO |
| 9. Do you often trim your nails? | Yes | NO |
| 10. Does the school provide education on water, sanitation, and hygiene (WASH)? | Yes | NO |

Thank you

References

1. Berhe, A. A., Aregay, A. D., Abreha, A. A., Aregay, A. B., Gebretsadik, A. W., Negash, D. Z., Gebreegziabher, E. G., Demoz, K. G., Fenta, K. A., & Mamo, N. B. (2020). Knowledge, Attitude, and Practices on Water, Sanitation, and Hygiene among Rural Residents in Tigray Region, Northern Ethiopia. *Journal of Environmental and Public Health*, *2020*, 1–9. <https://doi.org/10.1155/2020/5460168>
2. Vishnupriya, S., Prasad, S., Kasav, J. B., Trout, K., Murthy, S., Surapaneni, K. M., & Joshi, A. (2014). Water and sanitation hygiene knowledge, attitudes and practices among school settings in rural Chennai. *Journal of Water, Sanitation and Hygiene for Development*, *5*(2), 192–200. <https://doi.org/10.2166/washdev.2014.052>
3. Gebreeyessus, G. D., & Adem, D. B. (2018). Knowledge, Attitude, and Practice on Hygiene and Morbidity Status among Tertiary Students: The Case of Kotebe Metropolitan University, Addis Ababa, Ethiopia. *Journal of Environmental and Public Health*, *2018*, 1–9. <https://doi.org/10.1155/2018/2094621>
