# Supplementary material for: Knowledge, attitudes, and practices of safe drinking water, sanitation, and hygiene among pre-adolescent students in public and private schools in Jordan: cross-sectional school-based study
Source: Front Public Health. 2026 Sep 8;14:1925764. doi: 10.3389/fpubh.2026.1925764 (PMC13593898; doi:10.3389/fpubh.2026.1925764)
Supplement: Supplementary file 2 [file Table_2.docx]

**Tables Supplementary**

**Table S1. Knowledge items related to WASH**

| **Questions** | **Public**  **n**  **(%)** | **Private n**  **(%)** | **Overall n**  **(%)** | **p-value** |
| --- | --- | --- | --- | --- |
| **Knowledge** | | | | |
| **Know unsafe water causes diarrheal diseases** | **231 (38.5%)** | **227 (37.8%)** | **458 (76.3%)** | **0.701** |
| **Know water can get contaminated** | **273**  **45.5%** | **256**  **42.7%** | **529**  **88.2%** | **0.032** |
| **Know clean water sources should be used for hand washing** | **267**  **44.5%** | **263**  **43.8%** | **530**  **88.3%** | **0.611** |
| **Know the importance of hand soap** | **203**  **33.8%** | **246**  **41.0%** | **449**  **74.8%** | **0.000*** |
| **Knowledge of the consequences of not washing hands** | **257**  **42.8%** | **259**  **43.2%** | **516**  **86.0%** | **0.453** |
| **Know liquid wastes expose to disease** | **255**  **42.5%** | **262**  **43.7%** | **517**  **86.2%** | **0.408** |
| **Know animal dung causes diseases** | **230**  **38.3%** | **214**  **35.7%** | **444**  **74.0%** | **0.136** |
| **Know drinking clean water is considered a prevention mechanism for acute watery diarrhea** | **191**  **31.8%** | **196**  **32.7%** | **387**  **64.5%** | **0.670** |
| **Know sharing drinking cups without washing them brings health problem** | **234**  **39.0%** | **248**  **41.3%** | **482**  **80.3%** | **0.150** |
| **Knowing hand hygiene is considered a prevention mechanism for acute watery diarrhea** | **151**  **25.2%** | **137**  **22.8%** | **288**  **48.0%** | **0.253** |
| **Knowing using toilets and Proper Sanitation is considered a prevention mechanism for acute watery diarrhea** | **183**  **30.5%** | **180**  **30.0%** | **363**  **60.5%** | **0.802** |
| **Knowing toilets is essential and obligatory for every school** | **256**  **42.7%** | **269**  **44.8%** | **525**  **87.5%** | **0.109** |
| **Received information on WASH in the last 6 months** | **134**  **22.3%** | **175**  **29.2%** | **309**  **51.5%** | **<0.001*** |
| **The information received on WASH about Water quality** | **112**  **18.7%** | **158**  **26.3%** | **270**  **45.0%** | **<0.001*** |
| **The information received on WASH about proper toilet use and sanitation** | **103**  **17.2%** | **113**  **18.8%** | **216**  **36.0%** | **0.395** |
| **The information received on WASH about Hand hygiene** | **134**  **22.3%** | **182**  **30.3%** | **316**  **52.7%** | **<0.001*** |
| **Know human feces contain germs that can cause infection** | **201**  **33.5%** | **223**  **37.2%** | **424**  **70.7%** | **0.049*** |
| **Knowing both washing hands after a meal and before the meal is important** | **162**  **27.0%** | **204**  **34.0%** | **366**  **61.0%** | **<0.001*** |

**Table S2. Collinearity Statistics**

| **Variables** | **Tolerance** | **VIF** |
| --- | --- | --- |
| **First Step** | | |
| **Gender** | 1.000 | 1.000 |
| **School type** | .917 | 1.090 |
| **Age** | .999 | 1.001 |
| **Family #** | .918 | 1.089 |
| **Second Step** | | |
| **Gender** | .997 | 1.003 |
| **School type** | .912 | 1.096 |
| **Age** | .951 | 1.052 |
| **Family #** | .916 | 1.091 |
| **Knowledge** | .945 | 1.059 |
| **Third Step** | | |
| **Gender** | .995 | 1.005 |
| **School type** | .828 | 1.208 |
| **Age** | .941 | 1.063 |
| **Family #** | .914 | 1.094 |
| **Knowledge** | .637 | 1.569 |
| **Attitudes** | .586 | 1.707 |
